# St. Mary's and the Working Classes

**Published:** 1887-09-24

**Authors:** Thomas Ryan

**Affiliations:** Secretary


### St. Mary's and the Working Classes.

By Thomas Ryan, Secretary.

A Deputation Committee of the Sons of the Western District Amalgamated Temperance and other Friendly Societies called upon the governors of St. Mary's Hospital on Friday last, to present a sum of twenty-five pounds ten shillings, the outcome of a Sunday demonstration in aid of the hospital. They were met by several governors, and conducted round some of the wards, in the absence of the secretary, by Colonel Stanley G. Bird, one of the house visitors.

It is most pleasing to the authorities that societies of working-men should unite in this way to assist an institution, the value of which is known by personal experience to many among the Society's own members. It is gratifying to find that the experience of those whose misfortune, and at the same time good fortune, it has been to be the subjects of treatment within the hospital wards, is such as to lead them to contribute, and to induce others to contribute, in aid of its funds. Not less satisfactory is it that societies of this description should bring their donations personally, and meet the governors or the secretary, and in this way create a relationship, as it were, between the hospital and their own societies, which are labouring in a different branch of the same cause, namely, the amelioration of the condition of our fellow-man.

It has been very noticeable in deputations of this kind that there has been one member at least who has been a patient in the hospital—possibly selected on this account. It is the good fortune of this hospital to be frequently visited and assisted by societies of working-men, and there is not a feature of its daily experience that is more pleasing to those interested in it.

